# Matching Patients to Clinical Trials with Large Language Models

**Published:** 2023-07-28

**Authors:** Qiao Jin, Zifeng Wang, Charalampos S. Floudas, Jimeng Sun, Zhiyong Lu

**Affiliations:** 1 National Center for Biotechnology Information, National Library of Medicine, National Institutes of Health; 2 Department of Computer Science, University of Illinois Urbana-Champaign; 3 Center for Immuno-Oncology, Center for Cancer Research, National Cancer Institute, National Institutes of Health

## Abstract

Clinical trials are vital in advancing drug development and evidence-based medicine, but their success is often hindered by challenges in patient recruitment. In this work, we investigate the potential of large language models (LLMs) to assist individual patients and referral physicians in identifying suitable clinical trials from an extensive selection. Specifically, we introduce TrialGPT, a novel architecture employing LLMs to predict criterion-level eligibility with detailed explanations, which are then aggregated for ranking and excluding candidate clinical trials based on free-text patient notes. We evaluate TrialGPT on three publicly available cohorts of 184 patients and 18,238 annotated clinical trials. The experimental results demonstrate several key findings: First, TrialGPT achieves high criterion-level prediction accuracy with faithful explanations. Second, the aggregated trial-level TrialGPT scores are highly correlated with expert eligibility annotations. Third, these scores prove effective in ranking clinical trials and exclude ineligible candidates. Our error analysis suggests that current LLMs still make some mistakes due to limited medical knowledge and domain-specific context understanding. Nonetheless, we believe the explanatory capabilities of LLMs are highly valuable. Future research is warranted on how such AI assistants can be integrated into the routine trial matching workflow in real-world settings to improve its efficiency.

## Introduction

Clinical trials examine the effectiveness of medical interventions and provide crucial evidence that can be used to guide clinical practice. They also offer an opportunity for participants to receive experimental treatments that could potentially improve their health outcomes. However, matching patients to suitable clinical trials can be a challenging process^1–3^. This process includes analyzing a patient’s medical history, understanding the eligibility criteria of each clinical trial, and ensuring a match that satisfies both patient needs and trial requirements.

Manually matching patients and clinical trials is often labor-intensive, time-consuming, and prone to human errors. In recent years, artificial intelligence (AI) has shown promise in improving the efficiency and accuracy of patient-trial matching^4^. Based on the search directionality, there are two types of patient-trial matching tasks. The “trial-to-patient” scheme matches one trial to a list of candidate patients, which is a common need for clinical trial organizers and can be done by converting the trial criteria to structured query languages and searching the patient database^5–7^. On the other hand, the “patient-to-trial” scheme matches one patient to a list of candidate clinical trials^8–11^. In this study, we focus on the patient-centric “patient-to-trial” scheme because such a model can empower individual patients as well as referral offices to explore a large set of potentially eligible clinical trials. The core challenges lie in the heterogeneity and ambiguity inherent in patient records that induce significant challenges in natural language understanding.

A series of prior efforts encoded patient records and trial criteria text into dense embeddings using neural networks. The core aim is to project patient and trial inputs to the same embedding space with supervised training on paired patient-trial samples, thus enabling patient-trial matching through similarity search^12–14^. Nonetheless, these approaches suffer from the data-hungry nature of deep learning: endowing neural networks with the language understanding capability of ambiguous criteria texts and diverse patient records requires large training data. This is often infeasible because of lacking accessible paired patient health records and clinical trials. Besides, the black-box dense retrieval process is not explainable, and it is hard to debug when extrapolating it to previously unseen criteria and patient characteristics.

In this study, we evaluate how recent generative large language models (LLMs) can aid the process of patient-to-trial matching in a data-efficient and transparent way. LLMs are transformer-based sequence models^15^ with billions of parameters that can understand a given context and generate human-like text responses accordingly. LLMs are first pre-trained to predict the next token (language modeling) on a large corpus with trillions of words, which results in base models such as GPT-3^16^, PaLM^17^, and LLaMA^18^. Then, these base models are further fine-tuned to better follow human instructions for various purposes^19,20^, leading to assistant models like ChatGPT^21^. LLMs have demonstrated the capability of learning to solve new tasks at inference time when equipped with the prompting context, namely in-context learning (ICL)^16^. It has been shown that ICL makes LLMs excel on many natural language processing (NLP) tasks^22,23^, as well as biomedical tasks such as literature retrieval^24^, question answering^25–30^ and clinical trial design^31^. However, it was also observed that LLMs usually produce hallucinations with plausible-sounding but incorrect outputs^32^. It is hence crucial to offer evidence-grounded generated texts with human-readable rationales for high-stake biomedical domain-specific applications^33^. A pilot study has explored using LLMs to enhance the first-stage retrieval of clinical trials through information extraction^34^, while this study pivots on the fine-grained ranking and rationalizing the criteria-level patient-trial matching results using LLMs.

**Figure 1.**
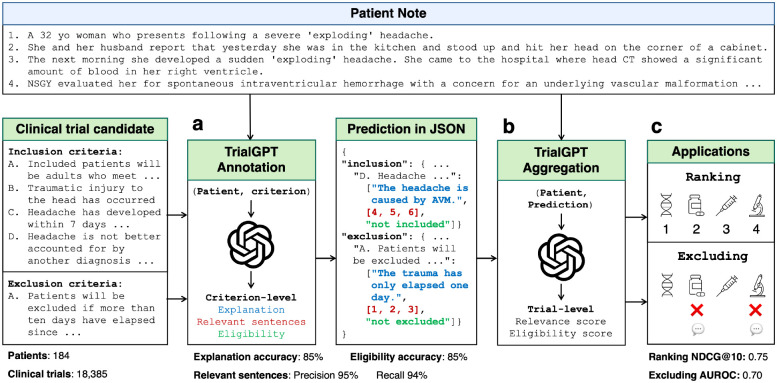
The overall architecture of TrialGPT. **(a)** Given a patient note and a clinical trial candidate as input, TrialGPT first uses LLMs to generate the relevance explanation, relevant sentence locations, and the patient eligibility for each criterion. **(b)** TrialGPT then uses LLMs to aggregate criterion-level predictions into trial-level scores. **(c)** The trial-level scores can be used to rank or exclude a list of clinical trial candidates for the given patient.

In this paper, we leverage LLMs to perform patient-to-trial matching using in-context learning, namely TrialGPT. As shown in Figure 1, TrialGPT handles two challenging tasks: (a) predicting criterion-level eligibility with explanations, (b) aggregating the criterion-level predictions to generate a trial-level score for downstream applications. Specifically, given a patient note and a candidate clinical trial, TrialGPT predicts three elements for each eligibility criterion: (1) a natural language explanation showing the relevance of the patient to the criterion, (2) locations of relevant sentences in the patient note that are relevant to the target criterion, and (3) the eligibility classification indicating whether the patient meets this criterion. We manually evaluate TrialGPT on 415 of patient-criterion pairs, and the results show that TrialGPT can accurately explain patient-criterion relevance, locate relevant sentences, and predict criterion-level eligibility. Then, TrialGPT further aggregates the criterion-level predictions to yield trial-level scores for ranking clinical trials. We evaluate these trial-level scores by TrialGPT on three publicly available cohorts of 184 patients and 18,238 manually annotated clinical trials. Experimental results show that the aggregated-TrialGPT scores are highly correlated with expert eligibility annotations, which can be used for effectively matching eligible trials with patients and excluding ineligible trials.

In summary, we present TrialGPT, a first-of-its-kind architecture that uses large language models to perform patient-trial matching. Our systematic evaluations show that TrialGPT can accurately predict criterion-level eligibility with faithful explanations. We also explore different pooling methods to aggregate the criterion-level predictions to trial-level scores that can be used to rank or exclude a list of candidate trials. As such, we anticipate that TrialGPT can be a valuable tool in assisting the process of patient-trial matching.

## Results

### Cohort characteristics

To evaluate TrialGPT, we use the patient notes and clinical trials with manual eligibility annotations from three publicly available cohorts: a test collection for patient-trial matching published by Special Interest Group on Information Retrieval (SIGIR) in 2016^9^, and the 2021 and 2022 Clinical Trials (CT) tracks^8^ of the Text REtrieval Conference (TREC). For each patient, we sample at most 50 clinical trials for each eligibility category: the TREC CT cohorts have “eligible”, “excluded” (“ineligible”) and “irrelevant” trials, while the SIGIR cohort does not have the “excluded” trials. The baseline statistics of these patient cohorts are shown in Table 1. We use the combination of these three cohorts as the final evaluation corpus.

### TrialGPT achieves a high criterion-level prediction accuracy

**Figure 2.**
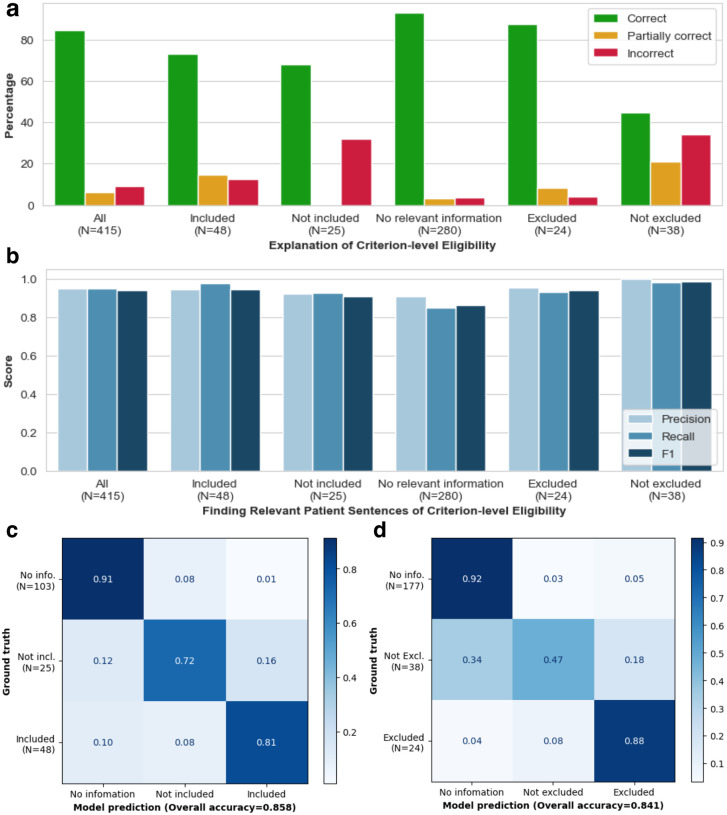
Manual evaluations of criterion-level predictions by TrialGPT. **(a)** The percentage of correct, partially correct, and incorrect relevance explanations generated by TrialGPT; **(b)** Evaluation results of the relevant sentences located by TrialGPT; **(c)** The confusion matrix of the TrialGPT-predicted eligibility for inclusion criteria; **(d)** The confusion matrix of the TrialGPT-predicted eligibility for exclusion criteria.

TrialGPT can annotate the criterion-level eligibility classification and offer the explanations and locations of relevant sentences, as shown in Figure 1a. In detail, it assigns each inclusion criterion a label within {Included, Not included, No relevant information} and exclusion criterion a label within {Excluded, Not excluded, No relevant information}. Then, it offers the rationales and identifies the evidence sentences. To evaluate this component, we manually annotate 415 criterion-level predictions of TrialGPT regarding three output elements: (1) the relevance explanation between the given patient note and the criterion, (2) the relevant sentence locations in the patient note, and (3) the criterion-level prediction of the given patient’s eligibility. The evaluation results are shown in Figure 2.

#### Evaluating relevance explanations:

We show the percentage of “correct”, “partially correct” and “incorrect” TrialGPT explanations in Figure 2a. Overall, most explanations are “correct” (84.6%) by manual evaluations, while only a small proportion of explanations are “partially correct” (6.27%) and less than 10% are “incorrect” (9.16%). We also found that most of the incorrect explanations are for criteria labeled as “not included” and “not excluded”, which usually require implicit inference as discussed in the case studies. TrialGPT exhibits much fewer mistakes when the criteria are explicitly “included” or “excluded”. Overall, the results suggest that TrialGPT can effectively explain how a patient is relevant to an eligibility criterion.

#### Evaluating relevant sentence locations:

We further compare the relevant sentences predicted by TrialGPT against the ground-truth expert annotations of relevant sentence locations. As shown in Figure 2b, the TrialGPT-predicted sentence locations are 94.9% correct (precision) and cover 93.8% of the ground-truth relevant sentence IDs (recall). Such high performance is consistent in breakdowns by eligibility. This shows that TrialGPT can faithfully locate relevant sentences in patient notes, which further improves the explainability of TrialGPT.

#### Evaluating eligibility prediction:

Finally, we evaluate the criterion-level eligibility labels predicted by TrialGPT with the ground-truth annotations. Figures 2c and 2d show the confusion matrices for these predictions. For the inclusion criteria, TrialGPT reaches a prediction accuracy of over 0.70 for all three labels, as indicated on the diagonal of the confusion matrix. For the exclusion criteria, while the accuracy is high for criteria labeled with “excluded” (0.88) and “no relevant information” (0.92), TrialGPT tends to misclassify the “not excluded” criteria as “no relevant information”. Overall, TrialGPT achieves an overall accuracy of 0.86 and 0.84 for the inclusion and exclusion criteria, respectively. This result suggests that TrialGPT can accurately predict patient eligibility at the criterion level.

### Aggregated TrialGPT scores correlate with trial-level eligibility

**Figure 3.**
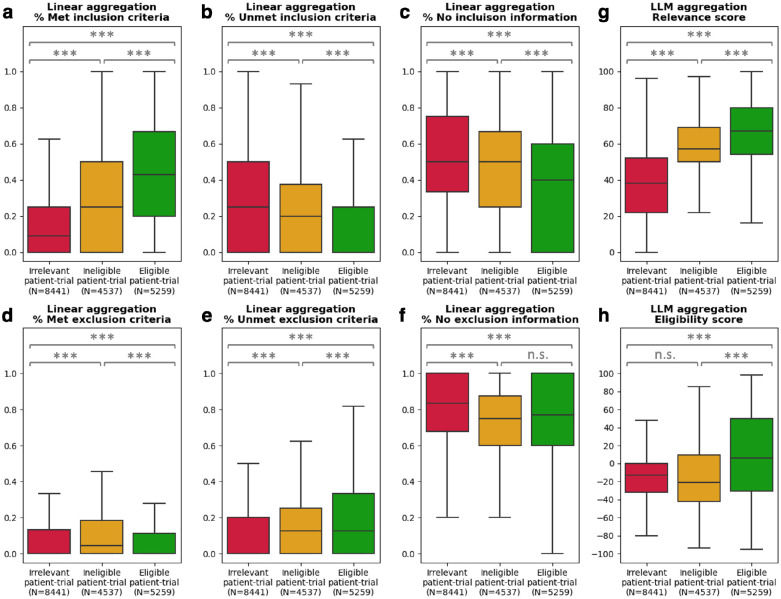
Correlation between differently aggregated TrialGPT scores and the ground-truth patient-trial eligibility labels. **(a)** The percentage of inclusion criteria predicted as “included” by TrialGPT; **(b)** The percentage of inclusion criteria predicted as “not included”; **(c)** The percentage of inclusion criteria predicted as “no relevant information”; **(d)** The percentage of exclusion criteria predicted as “excluded”; **(e)** The percentage of exclusion criteria predicted as “not excluded”; **(f)** The percentage of inclusion criteria predicted as “no relevant information”; **(g)** The LLM-aggregated relevance score; **(h)** The LLM-aggregated eligibility score. “***” denotes p < 0.001 and “n.s.” denotes not significant (p > 0.05) by independent t-test.

TrialGPT has been shown to achieve high prediction accuracy at the criterion level. However, since one clinical trial typically has many inclusion and exclusion criteria, trial-level scores should be computed as a way to decide the extent to which a given patient is eligible or ineligible. In this section, we evaluate how criterion-level predictions of TrialGPT can be aggregated into trial-level scores (component shown in Figure 1b). For this, we analyze the correlations between patient-trial eligibility and eight trial-level scores, which are computed by two types of methods: linear aggregations and LLM aggregations. The results are presented as box plots in Figure 3.

#### Linear aggregations:

Six scores are computed by counting the percentages of the criterion-level eligibility predictions of TrialGPT. Their correlations with trial-level eligibility labels are shown in Figure 3a–f.

- the percentage of inclusion criteria predicted as “included” by TrialGPT. As expected, Figure 3a implies that the patients meet the highest percentages of inclusion criteria in eligible clinical trials and meet the lowest percentages of inclusion criteria in the irrelevant clinical trials. The percentage of met inclusion criteria falls in between for relevant but ineligible trials;
- the percentage of inclusion criteria predicted as “not included”, which follows the reverse trends of the met inclusion criteria (Figure 3a);
- the percentage of inclusion criteria predicted as “no relevant information”. Figure 3c shows that patient notes contain higher percentages of relevant information for the inclusion criteria in the eligible trials;
- the percentage of exclusion criteria predicted as “excluded”. Interestingly, Figure 3d shows that patients meet more exclusion criteria in ineligible clinical trials than in irrelevant and eligible clinical trials, unlike other graphs that are either monotonically increasing or decreasing with regard to the irrelevant-ineligible-eligible order. This is a characteristic feature of patient-trial pairs that are explicitly excluded;
- the percentage of exclusion criteria predicted as “not excluded”. The percentages of unmet exclusion criteria follow the same trend as the percentage of the met inclusion criteria (Figure 3a), while less distinctive between groups;
- the percentage of exclusion criteria predicted as “no relevant information”, which shows that most exclusion criteria lack corresponding information in the patient notes.

#### LLM aggregations:

We also use LLMs to further aggregate the criterion-level predictions of TrialGPT, resulting in two scores:

- The general relevance score (0~100), where the irrelevant patient-trial pairs are much lower than the over two groups. Eligible and ineligible patient-trial groups have certain overlaps, but the former is still significantly higher than the latter;
- The eligibility score (−100~100), where negative scores denote ineligible, positive scores denote eligible, and a score of 0 denotes neutral. Overall, the eligible patient-trial pairs have the highest scores, and the ineligible patient-trial pairs have the lowest scores.

In summary, criterion-level TrialGPT predictions can be aggregated into trial-level scores that are highly correlated with patient-trial eligibility. The results of linear aggregations demonstrate that eligible patient-trial pairs have the highest proportions of met inclusion criteria and unmet exclusion criteria, while ineligible patient-trial pairs have the highest proportions of met exclusion criteria. In addition, the LLM aggregations are also significantly correlated with the manual eligibility labels. These results suggest that the aggregated scores of TrialGPT can be used to rank or exclude a list of candidate clinical trials for a given patient.

### TrialGPT can effectively rank and exclude candidate clinical trials

In this section, we evaluate TrialGPT on ranking a list of candidate clinical trials for a given patient (component Figure 1c). Based on the correlation analysis, we design a suite of scoring methods to aggregate criterion-level predictions of TrialGPT to generate a trial-level score for ranking the candidate trials. Table 2 shows the Normalized Discounted Cumulative Gain at rank 10 (NDCG@10) and Precision at rank 10 (P@10) of different methods in comparison to state-of-the-art models, which are described in Methods.

#### Ranking candidate clinical trials:

As shown in Table 2, TrialGPT outperforms all compared baselines, including dual-encoder, cross-encoder, and encoder-decoder models trained on different biomedical and clinical natural language inference (NLI)^47^ datasets. The best baseline for ranking clinical trials is the cross-encoder BioLinkBERT^45^ trained on MedNLI^41^, which achieves the NDCG@10 of 0.5558 and the P@10 of 0.4663. The most effective feature of TrialGPT for ranking is the LLM-aggregated relevance score. This feature alone achieves the NDCG@10 of 0.7421 and P@10 of 0.6705, which are much higher than other aggregations. Combining both linear and LLM aggregations yields the highest-ranking performance, with the NDCG@10 of 0.7480 and the P@10 of 0.6756.

#### Excluding ineligible clinical trials:

Table 2 also shows the Area Under the Receiver Operating Characteristic curve (AUROC) of excluding candidate trials, which is modeled as a binary classification task. The best baseline for excluding clinical trials is the dual-encoder SapBERT^44^ trained on MNLI^37^, SNLI^38^, SciNLI^39^, SciTail^40^, MedNLI^41^, and STSB^42^, achieving the AUROC of 0.5842. This result only shows marginal improvement over the random score baseline, indicating that the task of excluding ineligible trials presents significant challenges. Unlike in the task of ranking clinical trials, the percentage of inclusion criteria predicted as “not included” and the percentage of exclusion criteria predicted as “excluded” also achieve comparable AUROC individually. Again, TrialGPT outperforms all baselines, and the best TrialGPT feature is the LLM-aggregated score, and the optimal combination achieves an AUROC of 0.6954.

Overall, these experimental results show that TrialGPT can effectively rank candidate clinical trials and exclude ineligible clinical trials, which could potentially facilitate the trial-matching process.

### Case study and error analysis

Table 3 shows a case study of TrialGPT errors. In this case, the patient is a 32-year-old female who presented with intraventricular hemorrhage from an underlying Arteriovenous Malformation (AVM) after head trauma. The candidate trial (NCT04136860) studies long-term outcomes after different management strategies for high-level cerebral AVM. Evaluated by domain experts, TrialGPT makes two typical errors in this case: (1) Lack of medical knowledge. For the first inclusion criteria, TrialGPT notes that the patient has an AVM, but “it is not confirmed by DSA or MRI”. However, the patient is diagnosed by cerebral angiogram, which often denotes Digital Subtraction Angiography (DSA). One way to address this limitation is by adopting base models that possess more medical knowledge or connecting them with an external medical knowledge base. (2) Limited medical context understanding. TrialGPT also predicted “no relevant information” for the first exclusion criteria “Patients with multiple AVMs”. However, since the patient note contains the angiogram results, there would be mentions of multiple AVMs if the patient had them. Because there is no such mention, it is assumed that the patient only has one AVM. In addition, many clinical trials make pregnancy an exclusion criterion, which should be labeled as “not excluded” if the patient is male. However, TrialGPT labels such criteria as “no relevant information”. These examples show that TrialGPT can fail to capture medical domain-specific context dependency.

## Discussions

In this work, we present the first systematic evaluation of LLMs for matching patients and clinical trials. For this, we propose TrialGPT, a new architecture for patient-trial matching with LLMs. Evaluations on 415 patient-criterion pairs show that TrialGPT can accurately explain patient-criterion relation, locate relevant sentences in the patient note, and predict the eligibility labels at the criterion-level. Further evaluations on three publicly available cohorts of 184 patients and 18,238 annotated clinical trials show that the aggregated TrialGPT scores can be used to effectively rank and exclude candidate clinical trials. Our case study and analysis show that TrialGPT makes errors due to lack of medical domain-specific knowledge or context understanding capabilities from its base LLM (GPT-3.5), which might be improved with better base LLMs such as GPT-4.

The technical novelty of TrialGPT is to explore LLMs for two vital sub-tasks in clinical trial matching: (1) Criterion-level prediction: TrialGPT is able to match patient and clinical trials at criterion-level with explanations, where previous models suffer from the lack of annotated instances and cannot provide explanations; (2) Aggregation of criterion-level predictions: TrialGPT further utilizes LLMs to aggregate the criterion-level predictions to generate trial-level scores, which outperforms other linearly aggregated scores at ranking and excluding candidate clinical trials.

For evaluation, we use three publicly available patient-trial matching datasets, where the patients are represented by a paragraph of free-text clinical summary. However, clinical trial matching in real life often requires the recruiters to check the patients’ information more comprehensively, which involves longitudinal clinical notes, lab values, and even imaging data. This requires the model to (1) attend to much longer contexts, (2) process structured data, and (3) process multi-modal inputs. These aspects have not been evaluated by this study but are worth exploring in future work. We also notice that the overall task formulation of the SIGIR and TREC datasets might be over-simplified, as many trial restrictions are not considered (geolocations, recruitment status, etc.) and the eligibility annotation is not strict at handling criteria with no relevant information.

Last but not least, this work supports the position that the AI models for clinical trial matching should not be designed to replace human recruiters, but to empower them, and experts should always be in the loop of medical AI deployments^48^. As such, evaluation in real-life clinical trial matching scenarios should also focus more on efficiency improvement for human recruiters, instead of solely reporting the prediction performance. In this sense, we believe the explanation capability of TrialGPT, or more generally LLMs, to be particularly helpful. Prospective evaluation of how LLMs such as TrialGPT can assist human experts and improve their working efficiency for clinical trial matching remains an important future direction to explore.

## Methods

### Patient cohorts

We use three publicly available cohorts in this study: the SIGIR 2016 cohort, the TREC 2021 CT cohort, and the TREC 2022 CT cohort. Following the TREC CT tracks, we collect three eligibility labels for each patient-trial pair: (0) irrelevant, where the patient is “not relevant to the trial in any way”; (1) excluded, where the patient is explicitly excluded; and (2) eligible, where the patient is eligible to enroll in the trial. All three cohort annotations only use the patient note and eligibility criteria without considering the geolocations and recruitment status of the clinical trials.

#### SIGIR 2016:

The original cohort contains 60 patient case reports, but 1 report is removed since the report is about a group of patients (topic ID 201426, “A group of 14 humanitarian service workers…”). The patient notes are derived from the Clinical Decision Support (CDS) tracks in TREC 2014 and 2015, which are “medical case narratives created by expert topic developers that will serve as idealized representations of actual medical records”^49^. They typically describe the patient’s medical history, current symptoms, tests, eventual diagnosis, and treatments. Given a patient note, four medical assessors annotate each candidate clinical trial with three possible labels: (a) “would not refer this patient for this clinical trial”; (b) “would consider referring this patient to this clinical trial upon further investigation”; and (c) “highly likely to refer this patient for this clinical trial”. We consider the label a to be “irrelevant” and the label c to be “eligible”. Patient-trial pairs with the label b are not used in this work. The candidate clinical trials for annotation are derived from pooling various retrieval methods.

#### TREC 2021/2022 CT:

The TREC 2021 and 2022 CT tracks contain 75 and 50 patients, respectively. These patient notes are synthetic patient case descriptions, “such as what may be included as part of an admission note”. For each patient, they annotate three eligibility labels for the candidate clinical trial: irrelevant, excluded, and eligible. The candidate clinical trials are pooled from the submission systems of TREC participants.

### TrialGPT

TrialGPT is an architecture for patient-trial matching with large language models. It is composed of three modules: 1) a backbone LLM, 2) a criterion-level prediction module, and 3) a trial-level aggregation module. Here we denote the LLM as 𝒢, a patient note as a list of *P* sentences [*s*_1_, *s*_2_, …, *s*_*P*_], a clinical trial as composed of the background information B (containing the title, conditions, interventions, and the brief summary), a list of *M* inclusion criteria [*i*_1_, *i*_2_, …, *i*_*M*_], and a list of *N* exclusion criteria [*e*_1_, *e*_2_, …, *e*_*N*_].

### Backbone LLM

TrialGPT is LLM-agnostic, meaning that it can be plugged into different backbone LLMs. In this study, we use the GPT-3.5 API (model index: gpt-3.5-turbo, 0301 version, 4k context length) through Microsoft Azure’s OpenAI services. We set the inference temperature to 0 for deterministic outputs.

### Criterion-level prediction

The objective of this module is to output a free-text relevance explanation *R*, a list of relevant sentence IDs *S*, and the eligibility prediction *E* for each criterion based on the input patient note. For an inclusion criterion,

E∈{included, not included, no relevant information},

while for an exclusion criterion,

E∈{excluded, not excluded, no relevant information}.

We use different label sets for inclusion and exclusion criteria because the latter are often ambiguous. For example, exclusion criteria of “Pregnancy”, “The patient should not be pregnant”, “Pregnant patients will be excluded”, serve for the same purpose. Traditional entailment labels might not be suitable to distinguish the semantic differences, while our eligibility-oriented label sets provide an end-to-end solution.

As such, we make two LLM inference calls for each patient-trial pair: one for all inclusion criteria, and another one for all exclusion criteria. We use one-shot in-context learning^16^ where the exemplar is manually annotated and used across all test instances. Overall, the prompt includes the task description, the one-shot exemplar, the clinical trial background information B, and the inclusion ([*i*_1_, *i*_2_, …, *i*_*M*_]) or exclusion ([*e*_1_, *e*_2_, …, *e*_*N*_]) criteria. Motivated by chain-of-thought prompting^50^, we prompt the model to first generate the relevance explanation as grounding for future predictions of the relevant sentence IDs and the eligibility labels. In addition, we also prompt the model to generate criterion-level predictions in the JSON format, which can be easily parsed for aggregations. The TrialGPT prompt is shown in Table 4.

### Trial-level aggregation

After getting the criterion-level predictions, TrialGPT then aggregates such scores to generate a trial-level score that can be used for practical applications such as ranking and excluding clinical trials. Specifically, we denote the eligibility predictions of TrialGPT for the inclusion criteria and exclusion criteria as [*E*(*i*_1_), *E*(*i*_2_), …, *E*(*i*_*M*_)] and [*E*(*e*_1_), *E*(*e*_2_), …, *E*(*e*_*N*_)], respectively.

#### Linear aggregation:

six scores are simply derived based on the percentages of different eligibility predictions. While more sophisticated scoring methods can be used, we intentionally use these simple and linear aggregation strategies for better probing the capabilities of LLMs. For a trial’s inclusion criteria:

$$\%\text{ met inclusion criteria}=\frac{|\left\{E\left(i_{x}\right)=\text{included}|x=1,2,\ldots ,M\right\}|}{M}$$

$$\%\text{ unmet inclusion criteria}=\frac{|\left\{E\left(i_{x}\right)=\text{ not included}|x=1,2,\ldots ,M\right\}|}{M}$$

$$\%\text{ no relevant information about inclusion criteria}=\frac{|\left\{E\left(i_{x}\right)=\text{ no relevant information}|x=1,2,\ldots ,M\right\}|}{M}$$

For a trial’s exclusion criteria:

$$\%\text{ met exclusion criteria}=\frac{|\left\{E\left(e_{y}\right)=\text{excluded}|y=1,2,\ldots ,N\right\}|}{N}$$

$$\%\text{ unmet exclusion criteria}=\frac{|\left\{E\left(e_{y}\right)=\text{not excluded}|y=1,2,\ldots ,N\right\}|}{N}$$

$$\%\text{ no relevant information about exclusion criteria}=\frac{|\left\{E\left(e_{y}\right)=\text{no relevant information}|y=1,2,\ldots ,N\right\}|}{N}$$

#### LLM aggregation:

In addition, TrialGPT also uses LLMs to aggregate the criterion-level predictions by the prompt shown in Table 6. The generated scores are directly used as aggregation scores. Specifically, we consider two main features: general relevance and eligibility: The general relevance (*R*) indicates how relevant a patient is to clinical trial, while the eligibility score (*S*) denotes how eligible the patient is to the clinical trial. We restrict that:

0≤R≤100

where 0 indicates that the patient is irrelevant to the clinical trial and 100 suggests that the patient is exactly relevant to the clinical trial. We further restrict that:

−R≤S≤R

based on the assumptions that the absolute value of eligibility cannot be higher than relevance. We adopt self-consistency prompting^51^ with five calls and use the average scores.

#### Optimal combination:

We further combine the linear and LLM aggregation features, generating the optimal scores for ranking and excluding:

combination(ranking) =% met inclusion criteria +% LLM general relevance  + % LLM eligibility score

where combination(ranking) denotes the score used for ranking candidate clinical trials, and higher combination(ranking) suggests higher probability of being eligible. On the other hand, the optimal score for excluding ineligible clinical trials is:

combination(excluding)=I(% unmet inclusion criteria >0)+I(% met exclusion criteria >0) −% met inclusion criteria−% LLM general relevance −% LLM eligibility score

where I is an indicator function:

$$I(\text{condition})=\{\begin{array}{ll} 1, & \text{if condition is True}\\ 0, & \text{if condition is False} \end{array}$$

### Compared methods

The core of TrialGPT lies at the prediction of patient-criterion eligibility. These predictions are then aggregated for the ranking and excluding tasks. As such, we compare TrialGPT to a variety of pre-trained language models that can predict patient-criterion eligibility. Since there is no existing patient-criterion eligibility annotations for training a supervised model, we consider transfer learning from the biomedical NLI datasets. Specifically, we use three categories of baselines: dual-encoder, cross-encoder, and encoder-decoder.

Dual-encoder models are also known as bi-encoder, where the patient note and the criterion are separately encoded by pre-trained transformers, and the eligibility is modeled as the similarity of the encoding vectors:

$$\text{score}(\text{ranking})=\frac{\sum_{x=1}^{M}V{\left(\text{patient}\right)}^{T}V\left(i_{x}\right)}{M}-\frac{\sum_{y=1}^{N}V{\left(\text{patient}\right)}^{T}V\left(e_{y}\right)}{N}$$

$$\text{score}(\text{excluding})=\frac{\sum_{y=1}^{N}V{\left(\text{patient}\right)}^{T}V\left(e_{y}\right)}{N}$$

and:

$$V(\text{patient})=\text{Enc}\left(\left[s_{1},s_{2},\ldots ,s_{P}\right]\right)\in {\mathbb{R}}^{h}$$

$$V\left(i_{x}\right)=\text{Enc}\left(i_{x}\right)\in {\mathbb{R}}^{h}$$

$$V\left(e_{y}\right)=\text{Enc}\left(e_{y}\right)\in {\mathbb{R}}^{h}$$

where Enc denotes the pre-trained transformer encoder, and ℎ is the dimension of the vector representations.

Cross-encoder models take both the patient note and the criterion as input, which enables cross-attention computations between the tokens in both texts. The eligibility prediction is modeled as a 3-way classification task based on the special [CLS] embedding of BERT^52^. We use label space mapping functions *f* that maps an NLI label to an eligibility label:

$$E\left(i_{x}\right)=f_{\text{inc }}\left(\text{CrossEnc}\left(\left[s_{1},s_{2},\ldots ,s_{P}\right],i_{x}\right)\right)$$

$$E\left(e_{y}\right)=f_{\text{exc}}\left(\text{CrossEnc}\left(\left[s_{1},s_{2},\ldots ,s_{P}\right],e_{y}\right)\right)$$

where

$$f_{\text{inc}}(l)=\{\begin{array}{c} \text{included},\text{ if }l=\text{ entailment }\\ \text{not included},\text{ if }l=\text{ contradiction }\\ \text{no relevant information},\text{ if }l=\text{ neutral } \end{array}$$

and

$$f_{\text{exc}}(l)=\{\begin{array}{c} \text{excluded},\text{ if }l=\text{ entailment }\\ \text{not excluded},\text{ if }l=\text{ contradiction }\\ \text{no relevant information},\text{ if }l=\text{ neutral } \end{array}$$

Then we compute the combination scores based on the criterion-level prediction, similar to the optimal combination strategy used by TrialGPT:

combination(ranking)=% met inclusion criteria −% unmet inclusion criteria −% met exclusion criteria +% unmet exclusion criteria 

combination(excluding) =I(% unmet inclusion criteria >0)+I(% met exclusion criteria >0)−% met inclusion criteria 

Encoder-decoder models also take both the patient note and the criterion as input to the encoder, but instead of outputting a classification prediction, they generate the predicted NLI labels, e.g., “entailment”, “contradiction”, or “neutral”. These NLI labels are then mapped to eligibility labels that will be aggregated into the combination scores by the same methods described above for cross-encoder models.

### Evaluation settings

We report the NDCG@10 and P@10 for ranking candidate clinical trials, and AUROC for excluding ineligible clinical trials.

For computing NDCG@10 and P@10, we denote the ranked list of clinical trials as [*c*_1_, *c*_2_, …, *c*_*T*_], where *T* is the number of considered candidates. Their relevance scores are denoted as [*r*_1_, *r*_2_, …, *r*_*T*_], which are converted following the settings of the TREC Clinical Trials tracks:

$$r_{i}=\{\begin{array}{c} 0,\text{ if }E\left(c_{i}\right)=\text{irrelevant }\\ 1,\text{ if }E\left(c_{i}\right)=\text{ineligible }\\ 2,\text{ if }E\left(c_{i}\right)=\text{eligible } \end{array}$$

NDCG@10 is a measurement ranking quality, which is computed by:

$$\text{NDCG}@k=\frac{\text{DCG }@k}{\text{IDCG}@}$$

where

$$\text{DCG}@k=\overset{k}{\underset{x=1}{\sum }}\frac{r_{x}}{{\text{log}}_{2}(i+1)}$$

and

$$\text{IDCG}@\;k=\overset{k}{\underset{x=1}{\sum }}\frac{r_{\;x}^{\prime }}{{\text{log}}_{2}(i+1)}$$

where [*r*′_1_, *r*′_2_, …, *r*′_*T*_] denotes the relevance of an ideal ranking.

P@10 is another metric for ranking quality, computed by:

$$\text{P}@k=\frac{\sum_{x=1}^{k}r_{x}}{\text{max}(ℛ)\times k}$$

where ℛ denotes the set of relevance labels.

We draw the receiver operating characteristic (ROC) curve and compute the area under the ROC curve (AUROC) values using the sklearn package in Python.

## Figures and Tables

**Table 1.** Baseline statistics of the three patient cohorts used in this work. We show the mean ± standard deviation for applicable variables.

Cohort	SIGIR 2016	TREC 2021 CT	TREC 2022 CT
N	59	75	50
Age	38.3 ± 23.5	41.6 ± 19.4	35.3 ± 20.2
Gender (male: female)	29: 30	38: 37	28: 22
Note length (words)	88.1 ± 36.8	156.2 ± 45.4	109.9 ± 21.6
Note length (sentences.)	5.2 ± 2.1	9.5 ± 3.0	8.1 ± 1.5
Eligible trials / patient	6.5 ± 6.2	40.8 ± 13.8	38.3 ± 15.7
Excluded trials / patient	0.0	40.0 ± 14.8	32.7 ± 18.6
Irrelevant trials / patient	39.9 ± 11.3	50.0	50.0

**Table 2.** Performance of different methods for ranking and excluding clinical trials. The Sign() function assigns suitable signs for the corresponding task, e.g., for “% Included”, it will be “+” for ranking and “−” for excluding clinical trials.

Application			Ranking clinical trials		Excluding clinical trials
Method / Metric			NDCG@10	P@10	AUROC
Random score			0.3859	0.3629	0.4954
BioBERT^35^ (dual-encoder)		Further trained^36^ on MNLI^37^, SNLI^38^, SciNLI^39^, SciTail^40^, MedNLI^41^, and STSB^42^	0.4622	0.4236	0.5806
PubMedBERT^43^ (dual-encoder)			0.4824	0.4421	0.5804
SapBERT^44^ (dual-encoder)			0.4651	0.4424	0.5842
BioLinkBERT^45^ (cross-encoder)		Further trained on MedNLI^41^	0.5558	0.4663	0.5522
SciFive^46^ (encoder-decoder)		Further trained on MedNLI^41^	0.4461	0.3527	0.5591
TrialGPT criterion predictions	Linear aggr.	Sign(% Included)	0.6843	0.6044	0.6538
		Sign(% Not included)	0.5173	0.4861	0.6200
		Sign(% Excluded)	0.4010	0.3812	0.5910
		Sign(% Not excluded)	0.5119	0.4419	0.5106
	LLM aggr.	Sign(Relevance)	0.7421	0.6705	0.6650
		Sign(Eligibility)	0.7257	0.6533	0.6445
	Optimal feature combination		**0.7480**	**0.6756**	**0.6954**

**Table 3.** A case study of errors in the TrialGPT predictions (highlighted in red, with the comment and right answer in *italics blue*).

Patient note sentence (from patient 3 in cohort TREC 2021 CT)			Sent. ID
A 32 yo woman who presents following a severe ‘exploding’ headache.			0
She and her husband report that yesterday she was in the kitchen and stood up and hit her head on the corner of a cabinet.			1
The next morning she developed a sudden ‘exploding’ headache.			2
She came to the hospital where head CT showed a significant amount of blood in her right ventricle.			3
NSGY evaluated her for spontaneous intraventricular hemorrhage with a concern for an underlying vascular malformation.			4
Cerebral angiogram was done which showed abnormal vasculature with a draining vein from L temporal lobe penetrating deep white matter consistent with AVM.			5
The patient did continue to have a headaches but they were improving with pain medication.			6
The patient refused PT evaluation but was ambulating independently without difficulty.			7
She was discharged to home with her husband on [**2155-12-6**].			8

Trial: NCT04136860	TrialGPT predictions		
Inclusion criteria	Explanation	Relevant IDs	Eligibility
The diagnosis of AVM was confirmed with digital subtraction angiography (DSA) and/or magnetic resonance imaging(MRI)	There is relevant information that the patient has an AVM, but it is not confirmed by DSA or MRI. *(“Angiogram” is DSA)*	[5]	Not included *(Included)*
The SM grade was IV and V	There is relevant information that the patient has an AVM, but there is no information about the SM grade.	[5]	No relevant information
Exclusion criteria	Explanation	Relevant IDs	Eligibility
Patients with multiple AVMs	There is no relevant information of multiple AVMs in the patient note.	[] *([5] suggests one AVM)*	No relevant information *(Not excluded)*
Patients with hereditary hemorrhagic telangiectasia (HHT)	There is no relevant information of HHT in the patient note.	[]	No relevant information
Patients with missing clinical and imaging data	There is no relevant information of missing clinical and imaging data in the patient note.	[]	No relevant information

**Table 4.** An example prompt for TrialGPT to predict criterion-level eligibility. The {one_shot_exemplar} is shown in Table 5. {patient_note} and {clinical_trial} denote the input patient note text and the clinical trial criteria, respectively.

Hello. You are a helpful assistant for clinical trial recruitment. Your task is to compare a given patient note and the inclusion criteria of a clinical trial to determine the patient’s eligibility. The factors that allow someone to participate in a clinical study are called inclusion criteria. They are based on characteristics such as age, gender, the type and stage of a disease, previous treatment history, and other medical conditions. You should check the inclusion criteria one-by-one, following the steps below: 1. For each inclusion criterion, first think step-by-step to explain if and how the patient note is relevant to the criterion. You should explain in detail if there is relevant information. 2. Then, if there is relevant information, you must annotate a list of relevant sentence IDs of the patient note. If there is no relevant information, you must annotate an empty list. 3. Finally, annotate the patient eligibility for this specific inclusion criterion: the eligibility must be ‘no relevant information’ if there is no relevant information. Otherwise, the patient can only be ‘included’ or ‘not included’ if there are relevant sentences. ‘included’ means that the patient meets the inclusion criterion, and ‘not included’ means that the patient contradicts the inclusion criterion. 4. You should output only a JSON dict exactly formatted as: dict{str(inclusion_criterion): list[str(relevance_explanation), list[int(sentence_id)], str(‘included’|’not included’|’no relevant information’)]} {one_shot_exemplar} Here is the patient note, each sentence is led by a sentence_id: {patient_note} Here is the clinical trial: {clinical_trial} Plain JSON output without indent:

**Table 5.** One-shot exemplar used for in-context learning by TrialGPT to predict criterion-level eligibility. The

Here is an example patient note, each sentence is led by a sentence_id: 0. Patient is a 45-year-old man with a history of anaplastic astrocytoma of the spine complicated by severe lower extremity weakness and urinary retention s/p Foley catheter, high-dose steroids, hypertension, and chronic pain. 1. The tumor is located in the T-L spine, unresectable anaplastic astrocytoma s/p radiation. 2. Complicated by progressive lower extremity weakness and urinary retention. 3. Patient initially presented with RLE weakness where his right knee gave out with difficulty walking and right anterior thigh numbness. 4. MRI showed a spinal cord conus mass which was biopsied and found to be anaplastic astrocytoma. 5. Therapy included field radiation t10-l1 followed by 11 cycles of temozolomide 7 days on and 7 days off. 6. This was followed by CPT-11 Weekly x4 with Avastin Q2 weeks/ 2 weeks rest and repeat cycle. Here is an example clinical trial: Title: Is the Severity of Urinary Disorders Related to Falls in People With Multiple Sclerosis Target diseases: Fall, Multiple Sclerosis, Lower Urinary Tract Symptoms Interventions: Clinical tests Summary: Falls are a common problem in people with multiple sclerosis (PwMS) and can lead to severe consequences (trauma, fear of falling, reduction of social activities). Prevention of falls is one of the priority targets of rehabilitation for PwMS and walking difficulties, which can result of different factors (motor impairment, ataxia, sensitive disorders, fatigability…). Urinary incontinence has been evoked as predictive of falls. But lower urinary tract symptoms (LUTSs) are frequent in PwMS, the prevalence of LUTSs is high (32–96.8%) and increases with MS duration and severity of neurological deficiencies and disabilities. Overactive bladder (OAB) is the most common symptom. Despite its high prevalence and impact on quality of life, the severity of LUTSs has never been studied as specific risk factor of falling. However, urinary urgency and urinary incontinence could lead to precipitation and thus could increase the risk of falling in these patients.~The aim of the study was to assess the relationship between severity of LUTSs and risk of falling in PwMS.~Patients were asked about the number of falls in the past three months and in the past year, and the circumstances in which they occurred (frequency, home, outdoors, going to void, during urinary urgency, nocturia). Severity of LUTSs were assessed by the Urinary Symptoms Profile (USP) Score and patient were classified as with or without urinary incontinence. Number of micturition by night were specifically asked. To take into account motor difficulties and fear of falling, other clinical evaluations were done. The impact of MS on walking was assessed by the 12-Item Multiple Sclerosis Walking Scale (MSWS12) questionnaire, the Expanded Disability Status Scale score, and by clinical test with the Time to be Ready to Void (TRV). Fear of falling was assessed by a simple question and with Falls Efficacy Scale-International (FES-I) Questionnaire.~The primary aim was to assess the relationship between severity of LUTSs and occurrence of falls during the past 3 months. The primary outcome was the importance of overactive bladder (OAB) symptoms with OAB USP score. The secondary outcomes were the existence of urinary incontinence, the warning time (defined as the time from the first sensation of urgency to voiding or incontinence), the importance of nocturia and the other scores of USP questionnaire (low stream and stress urinary incontinence).~The secondary aims were to look for the relationship between severity of LUTSs and occurrence of falls during the past year, and to assess the relationship between falls and the classical risk factors of falls. Inclusion criteria: inclusion criteria: age ≥ 18 years Multiple sclerosis (MS) diagnosis Lower urinary tract symptoms with or without treatment, Expanded Disability Status Scale score between 1 and 6.5 Example plain JSON output without indent: {“age \u2265 18 years”: [“The patient is a 45-year-old man, older than 18.”, [0], “included”], “Multiple sclerosis (MS) diagnosis”: [“The patient has never been diagnosed with multiple sclerosis, so he does not meet the target condition of this trial.”, [0, 1, 2, 3, 4, 5, 6], “not included”], “Lower urinary tract symptoms with or without treatment”: [“The patient has urinary retention complication and has been treated.”, [0], “included”], “Expanded Disability Status Scale score between 1 and 6.5”: [“There is no relevant information of this score in the patient note.”, [], “no relevant information”]}

**Table 6.** TrialGPT aggregation prompt for generating the trial-level scores.

Hello. You are a helpful assistant for clinical trial recruitment. You will be given a patient note, a clinical trial, and the patient eligibility predictions for each criterion. Here is the patient note: {patient_note} Here is the clinical trial description: {clinical_trial} Here is the criterion-level eligibility prediction: {TrialGPT_criterion_predictions} Your task is to output two scores, a relevance score (R) and an eligibility score (E), between the patient and the clinical trial. First explain the consideration for determining patient-trial relevance. Predict the relevance score R (0~100), which represents the overall relevance between the patient and the clinical trial. R=0 denotes the patient is totally irrelevant to the clinical trial, and R=100 denotes the patient is exactly relevant to the clinical trial. Then explain the consideration for determining patient-trial eligibility. Predict the eligibility score E (-R~R), which represents the patient’s eligibility to the clinical trial. Note that -R<=E<= R (the absolute value of eligibility cannot be higher than the relevance), where E=-R denotes that the patient is totally ineligible (not included by any inclusion criteria, and excluded by all exclusion criteria), E=R denotes that the patient is totally eligible (included by all inclusion criteria, and not excluded by any exclusion criteria), E=0 denotes the patient is totally neutral (i.e., no relevant information for all inclusion and exclusion criteria). Finally, you should always repeat the R and E scores in the last line (first R, then E) by `R=?, E=?`, e.g., `R=75, E=−50`.

## Data Availability

The TREC Clinical Trial 2021 and 2022 cohorts can be downloaded from http://www.trec-cds.org/2021.html and http://www.trec-cds.org/2022.html, respectively. The SIGIR cohort is publicly available at https://data.csiro.au/collection/csiro:17152. We will release the code of TrialGPT upon publication.
